# Identifying the benefits of recovery programs of aquatic gymnastics, aquatic ludotherapy and therapeutic swimming on human motor, kinetotherapeutic and mental capacity

**DOI:** 10.1016/j.heliyon.2024.e38690

**Published:** 2024-09-29

**Authors:** Dana Badau, Adela Badau

**Affiliations:** a Faculty of Physical Education and Mountain Sports, Transilvania University of Brasov, Brasov, Romania; b Department SL2- Physical Education, “G.E. Palade” University of Medicine, Pharmacy, Sciences and Technology, Targu Mures, Romania

**Keywords:** Aqua exercises programs, Promoting health, Motor capacity, Mental capacity, Kinetotherapeutic rehabilitation, Aquatic gymnastics, Aquatic ludotherapy, Therapeutic swimming

## Abstract

**Introduction:**

This study aimed to evaluate the motor, kinetotherapeutic, and psychological benefits of three aquatic therapeutic programs—aquagym recovery (AG), aquatic ludotherapy (ALT), and therapeutic swimming (TS)—for students specializing in balneo-physio-kinetotherapy.

**Methods:**

The study group consisted of 101 students, out of which 43 students from the balneo-physiokinetotherapy study program (BFKT) and 67 master's degree students from physical therapy and functional rehabilitation (PTFR). The questionnaire was named Questionnaire regarding the benefits of therapeutic aquatic programs (QBTAP), and included 3 subscales according to the typology of the targeted objectives, namely: motor, kinetotherapeutic and psychological. The items of each subscale correspond to the objectives identified in the content of the 3 aquatic programs stipulated in the content of the courses corresponding to the disciplines in the education plans of academic study programs (BFK, TRFR). The motor subscale included 8 items, the kinetotherapeutic subscale included 16 items, and the psychological subscale included 10 items. The evaluation of the QBTAP questionnaire 5-point Likert scale. The three evaluated programs were: recovery aquagym (AG), aquatic ludotherapy (ALT) and therapeutic swimming (TS).

**Results:**

The results and conclusion sections should highlight the main findings succinctly. For example: "The QBTAP showed high internal consistency (Cronbach's Alpha >0.9) across all subscales and programs, with significant differences identified between the three programs (p < 0.05).

**Conclusion:**

The study concludes that aquagym most effectively improves motor capacity, aquatic ludotherapy enhances psychological function, and therapeutic swimming is most beneficial for recovery and rehabilitation.

## 1 Introduction

Performing different programs in the aquatic environment (recovery aquagym, aquatic play therapy and therapeutic swimming) creates major benefits for the health of the body, and their adaptation for prophylactic and therapeutic purposes influences the motor, functional and mental capacity of the practitioners. The diversification of the forms of practicing aquatic exercise programs is in a continuous dynamic as a result of the expansion of scientific knowledge and the diversification of the technologies and equipment used. In the present study, we want to highlight the most relevant motor, kinetotherapeutic and psychological benefits of practicing three forms of exercise in water: recovery aquagym, aquatic ludotherapy and therapeutic swimming. We consider that the identification of the most relevant benefits induced by the three aquatic exercise programs will allow the understanding of their impact on: health, harmonious development, prevention of physical deficiencies and health problems and especially on mental functions by combating stress and the negative effects of everyday life and professional life.

The interaction between physical and mental health is a major and current concern of specialists and requires a holistic approach to their benefits and therapeutic impact [1,2]. In this sense, the analysis of the motor, kinetotherapeutic and mental health benefits will facilitate the identification of the complexity of the benefits of therapeutic aquatic programs in the opinion of the specialist. Understanding how different aquatic therapeutic programs contribute to the improvement of physical and mental health will facilitate the optimization of the therapeutic process of recovery and rehabilitation depending on the patients' particularities and the specific pathology. Understanding the influence of therapeutic programs facilitates the personalization of therapeutic intervention protocols [3,4].

Aquagym is an optimal combination of: basic gymnastics and aerobic exercises, fitness, stretching and hydromassage, mainly aimed at perfecting physical development and improving motor and functional capacity [[5], [6], [7], [8]]. Analyzing aquagym in all its forms of practice, through the influences it has on: health, somatic and human personality dimensions (temperament, character, skills, intelligence and creativity) it can be stated that this kind of motor activity contributes to a very large extent, together with other forms of organizing physical activities, in shaping and contouring the human body and human personality [9,10]. Aquagym fits into the modern trends of practicing physical exercise by combining various, simple and complex actuation systems, adapted to the influences induced by the aquatic environment and by using sports materials for didactic purposes such as: wands, handhelds and special bags with sand made for the ankles, etc. [11,12]. The aquatic gymnastics are characterized according with the contact with the bottom of the pool by: *low impact*, *high impact* and *non-impact* involving those movements that are performed when the body is partially immersed, but the feet do not have contact with the bottom of the pool, in pools deeper than 1.60 m [[13], [14], [15], [16]]. The equipment and materials used in qaugim are adapted or identical to those in fitness and aerobics.

Aquatic ludotherapy includes any exercise integrated into a few easily accessible rules that can be turned into a game [17]. Aquatic recreational activities, used for prophylactic and therapeutic purposes, but especially corrective, influence both the shape and the structure of the human body, as well as the functional improvement of the body organs [18,19]. Performed systematically, the aquatic recreational activities can tone, in conditions of shortening, the hypotonic muscle groups, but also lengthen and relax the contracted ones, which allows the correction of physical deficiencies [20,21].

Physical and mental health is a major concern of specialists, and the therapeutic benefits of aquatic programs for therapeutic and prophylactic purposes focus on optimizing motor capacity, recovery and rehabilitation capacity and reducing the effects of stress and mental exhaustion and the risks of mental disorders. Performing movements in the water through therapeutic swimming, which is an adapted version of swimming with major benefits on health, the recovery and rehabilitation process, on mental functions, combating obesity, etc. [[22], [23], [24]]. As a result, the muscular effort is also less tiring, even having a toning effect. Therapeutic swimming has proven itself useful in many conditions such as: postural re-education and corrections of vertebral static disorders, recovery after surgical interventions with or without immobilization in bed; obesity, osteoporosis; recovery after orthopedic surgery (hip, knee, osteosynthesis) etc. [[25], [26], [27]].

Studies looking at the effects of practicing aquatic activities compared to the same movements performed on the ground, have indicated that the motor and functional responses are superior [[28], [29], [30]]. Other researchers claim that the effects due to the buoyancy of the aquatic environment on the body cause decreases in the axial load of the spine, which allows making certain movements, sometimes impossible to perform on the ground [31,32]. The aquatic physical exercise produces physiological reactions different from those produced on land [33,34]. This is due to the hydrostatic effects of water on the cardiac and respiratory systems as a reaction to the thermoregulatory process [35,36].

Analyzing the specialized literature, we have only partially identified studies that analyze the benefits of these types of aquatic programs on motor, kinetotherapeutic and psychological components. The specialized literature focused on qualitative therapeutic assessment of aquatic programs and did not address the impact of the benefits on physical and mental health: motor capacity, kinetotherapeutic and mental capacity. To carry out the study, we systematized the objectives of the 3 aquatic activities programs according to the components that aim to be optimized from the point of view of motor capacity, kinetotherapeutic and mental capacity. In this sense, we consider that the results of our study will contribute to the completion of the knowledge of the impact that these aquatic exercise programs have on the motor capacities, on the therapeutic recovery capacity and on the mental capacity from the perspective of the physiotherapy specialists and of the future physiotherapy specialists.

This study aimed to assess the impact of three aquatic therapeutic programs—recovery aquagym (AG), aquatic ludotherapy (ALT), and therapeutic swimming (TS)—on motor, kinetotherapeutic, and psychological outcomes in students specializing in balneo-physio-kinetotherapy. We hypothesized that the effectiveness of these programs is dependent on their impact on motor, therapeutic recovery, and mental capacitieson the impact of their benefits in terms of: motor, kinetotherapeutic and mental capacity.

## 2 Materials and methods

### 2.1 Participants

The study involved 101 students, with 43 enrolled in the balneo-physiokinetotherapy (BFKT) program and 67 in the master's program in physical therapy and functional rehabilitation (PTFR). The average age was 23.07 years, with 64 female (64.4 %) and 36 male (35.6 %) participants. Mediul de provenienta si de resedinta nu au influentat rezultatele studiului: 93 (97.1 %) urban, 8 (2.9 %) rural; mediul de resedinta 99(98 %) urban, 2 rural (2 %).

Inclusion criteria: active students, participation in the aquagym, aquatic ludotherapy and therapeutic swimming programs, full completion of the questionnaire.

### 2.2 Study design

The study was conducted in Targu Mures during 2022–2023, using a self-developed questionnaire (QBTAP) administered online. Participants had prior experience with all three aquatic programs as part of their academic curriculum. The collected results were statistically processed in accordance with the purpose of the study. The theoretical courses and practical works were implemented for the experimental group for the first time only during the study (both in the bachelor's program and in the master's program), the participants were not previously familiar with the three aquatic programs specific to the study. We mention that the three aquatic programs were implemented in the academic curriculum from the bachelor's and master's degrees only at the University of Targu Mures, at the other specialized faculties they are found only in the form of a single aquatic program from the three analyzed in the present study.

### 2.3 Measures

The QBTAP consisted of three subscales—motor, kinetotherapeutic, and psychological—corresponding to the objectives of the three aquatic programs: recovery aquagym (AG), aquatic ludotherapy (ALT), and therapeutic swimming (TS). Each subscale was assessed using a 5-point Likert scale. The motor subscale included 8 items, the kinetotherapeutic subscale included 16 items, and the psychological subscale included 10 items. The three evaluated programs were: recovery aquagym (AG), aquatic ludotherapy (ALT) and therapeutic swimming (TS). We counted the points given by the study subjects for each item of the 3 subscales of the questionnaire. The three aquatic programs were introduced for the first time in the year before the study at the undergraduate program, and the questionnaire was pre-tested in order to validate it on this previous generation in order to correct the statements and refine the questionnaire. In order to avoid biased answers, the subjects of the study were instructed to answer professionally, without taking into account the preferences for a certain type of program among the three aquatic programs included in the study.

### 2.4 Statistical analysis

Statistical analysis was performed using IBM SPSS Statistics 24. Key analyses included PCA for Bartlett's test and KMO, and ANOVA to assess differences between programs, with significance set at p < 0.05. Cronbach's alpha was used to evaluate internal consistency. The internal consistency of the questionnaire was calculated by the statistical parameter Cronbach's alpha with the following interpretation: >0.7 is acceptable, >0.8 is good and >0.9 is higher.

## 3 Results

In Table 1, it is highlighted that Cronbach's Alpha values for all 3 subscales (motor, kinetotherapeutic and mental), and for all types of aquatic programs (AG, ATL, TS), were higher than 0.9, which reflects a very high internal consistency. Also, the KMO values for all types of programs, for the 3 subscales of the questionnaire, were between 0.872 and 0.948, which indicates that the sampling was adequate. Bartlett's test of sphericity reflects that the correlation between items for each subscale was very broad for PCA, and the results were statistically significant reported at the reference value of 0.05. The ANOVA results highlight statistically significant differences (p value < 0.05) between the 3 aquatic exercise programs for each of the 3 subscales of the questionnaire applied in the study.

**Table 1:** Statical analysis of Cronbach's Alpha, KMO and Bartlett's Test and ANOVA of QBTAP of study.

Subscale	Aquatic program	Cronbach's Alpha	KMO and Bartlett's Test			ANOVA	
			KMO Sampling	Chi^2^ (df)	Sig.	F	p
Motor	AG	,938	,892	635,640 (28)	,000	5835	0,010
	ATL	,906	,872	373,375 (28)	,000		
	TS	,924	,905	526,377 (28)	,000		
Kineto-terapeutic	AG	,957	,934	1346,203 (120)	,000	5012	0,011
	ATL	,946	,929	1094,025 (120)	,000		
	TS	,961	,948	1387,490,120)	,000		
Psychological	AG	,937	,918	722,510 (45)	,000	6513	0,005
	ATL	,939	,892	788,263 (45)	0,000		
	TS	,921	,914	764,900 (45)	0,000		

AG – Recovery aquagym program, ATL - Aquatic ludotherapy program, TS - therapeutic swimming program, KMO Sampling - Kaiser-Meyer-Olkin Measure of Sampling Adequacy, Chi^2-^Approx. Chi-Square, df –degree of freedom, Sig-level of statistical significance.

Table 2 includes the number of subjects and the percentages of the mode of distribution of the results of the Motor Subscale included in the Questionnaire regarding the benefits of therapeutic aquatic programs. By analyzing the evaluation results of the recovery aquagym (AG) program, we notice that the largest number of respondents who gave 5 points on the Likert scale, compared to the other 2 programs, were concerned with: item 2 Improvement of effort capacity (43.6 %), item 3 body shaping (50.5 %), item 4 Muscle toning (52.5 %), item 8 improving body posture (52.5 %). Compared to the Aquagym program and the therapeutic swimming program, for the aquatic ludotherapy program (ATL) the percentages of respondents who gave 5 points, related to the highest frequency of responses, concerned the following items: 5. Improvement of psychomotor capacity (41.6 %), 6. Development of motor skills (38.6 %) and 7. Formation of motor skills (43.6 %). The therapeutic swimming program (TS), compared to the other two programs, registered the highest frequency of answers rated with the maximum score only for item 1 Harmonious physical development (47.5 %).

**Table 2:** The distribution of the subjects' options according to the score given on each item in the Motor Subscale of QBTAP.

Motor - Subscale – Likert Scale Points											
Items	Aquatic program	5 points		4 points		3 points		2 points		1 point	
		fi	%	fi	%	fi	%	fi	%	fi	%
1 Harmonious physical development	AG	41	40,6	34	33,7	18	17,8	7	6,9	1	1,0
	ATL	18	17,8	34	33,7	33	32,7	14	13,9	2	2,0
	TS	48	47,5	25	24,8	16	15,8	12	11,9	–	–
2 Improvement of effort capacity	AG	44	43,6	28	27,7	24	23,8	3	3,0	2	2,0
	ATL	16	15,8	30	29,7	29	28,7	23	22,8	3	3,0
	TS	33	32,7	23	22,8	31	30,7	12	11,9	2	2,0
3 Body shaping	AG	51	50,5	25	24,8	19	18,8	5	5,0	1	1,0
	ATL	13	12,9	23	22,8	29	28,7	31	30,7	5	5,0
	TS	32	31,7	29	28,7	19	18,8	20	19,8	1	1,0
4. Muscle toning	AG	53	52,5	24	23,8	21	20,8	2	2,0	1	1,0
	ATL	21	20,8	19	18,8	36	35,6	22	21,8	3	3,0
	TS	39	38,6	30	29,7	22	21,8	7	6,9	3	3,0
5. Improvement of psychomotor capacity	AG	26	25,7	45	44,6	23	22,8	5	5,0	2	2,0
	ATL	42	*41,6*	35	34,7	19	18,8	5	5,0	–	–
	TS	35	34,7	31	30,7	25	24,8	10	9,9	–	–
6. Development of motor skills	AG	36	35,6	40	39,6	18	17,8	6	5,9	1	1,0
	ATL	39	38,6	25	24,8	29	28,7	7	6,9	1	1,0
	TS	37	36,6	25	24,8	29	28,7	9	8,9	1	1,0
7 Formation of motor skills	AG	31	30,7	38	37,6	24	23,8	7	6,9	1	1,0
	ATL	44	43,6	27	26,7	25	24,8	4	4,0	1	1,0
	TS	36	35,6	31	30,7	26	25,7	8	7,9	–	–
8 Improving body posture	AG	53	52,5	26	25,7	14	13,9	7	6,9	1	1,0
	ATL	29	28,7	28	27,7	28	27,7	12	11,9	4	4,0
	TS	64	63,4	16	15,8	17	16,8	3	3,0	1	1,0

AG – Recovery aquagym program, ATL - Aquatic ludotherapy program, TS - therapeutic swimming program, fi -frequency.

The statistical processing showed in Table 3 allows us to observe that in the Motor Subscale the highest average score was recorded for the Recovery aquagym program (AG) with 32.59 (35 %) points, followed by the therapeutic swimming program with 31.36 (33.8 %) points and the lowest score of 29.03 (31.2 %) for Aquatic ludotherapy program. For the AG, the items that recorded the highest average score were: item 4. Muscle toning with 4.02 points and item 8 Improving body posture with 4.21 points, and the lowest score was recorded by items: 7 Training motor skills and 5. Improving psychomotor ability. In the Aquatic ludotherapy program, the highest average score was obtained by items: 5. Improving psychomotor ability with 4.12 points and 7. Training motor skills with 4.07 points, and the lowest average score by item 3. Body modeling with 3.07 points. The therapeutic swimming program recorded the highest average score for items: 8 Improving body posture with 4.37 points and for item 1 Harmonious physical development with 4.07 points, and the lowest score for item 3 Body shaping with 3.70 points.

**Table 3:** Descriptive statistics of the Motor Subscale of QBTAP for the three aquatic programs of the study.

Motor - Subscale									
Activities	AG			ATL			TS		
Item	Σ	X	SD	Σ	X	SD	Σ	X	SD
1. Harmonious physical development	410,00	4,05	,97	355,00	3,51	1,01	412,00	4,07	1,05
2. Improvement of effort capacity	412,00	4,07	,98	336,00	3,32	1,08	376,00	3,72	1,10
3. Body shaping	423,00	4,18	,97	311,00	3,07	1,11	374,00	3,70	1,14
4. Muscle toning	429,00	4,24	,92	336,00	3,32	1,12	398,00	3,94	1,07
5. Improvement of psychomotor capacity	391,00	3,87	,92	417,00	4,12	,89	394,00	3,90	,99
6. Development of motor skills	407,00	4,02	,93	397,00	3,93	1,02	391,00	3,87	1,04
7. Formation of motor skills	394,00	3,90	,95	412,00	4,07	,96	398,00	3,94	,96
8. Improving body posture	426,00	4,21	,99	369,00	3,65	1,13	442,00	4,37	,93
Total	3292	32,59	**-**	2933	29,03	**-**	3185	31,53	**-**
Percentage	35 %		**-**	31,2 %		**-**	33,8 %		**-**

AG – Recovery aquagym program, ATL - Aquatic ludotherapy program, TS - therapeutic swimming program, Σ - sum, X – mean, SD−standard deviation.

Analyzing the results presented in Table 4 of the QBTAP for the kinetotherapeutic subscale of the recovery aquagym (AG) program, we notice that the largest number of respondents who gave 5 points on the Likert scale, compared to the other 2 programs, targeted the most items: 2 Stimulates blood circulation (57.4 %), 3. Increases breathing capacity (54.5 %), 4. Increases immunity and hardens the body (40.5 %), 10. Maintains homeostatic balance (32.7 %), 11.Combating the effects of aging (33.4 %), 15. Improving metabolism (50.5 %) and 16. Combating obesity (67.3 %). For the aquatic ludotherapy program (ATL) compared to the other two programs, the highest percentage frequency of 5 points was recorded only in item 7. Muscle relaxation (33.7 %). The therapeutic swimming (TS) program, compared to the other two programs, recorded the highest frequency of responses appreciated with the maximum score to the items: 1. Optimizing the general state of health (50.5 %), 5. Improving joint mobility (56, 4 %), 6. Reduction of osteoporosis (39.6 %), 7. Muscle relaxation (33.7 %), 8. Postural correction and rehabilitation (65.3 %), 9. Improvement of motor control (45.5 %), 10. Maintaining homeostatic balance (32.7 %), 11. Combating the effects of aging (33.4 %), 12. Psychomotor recovery (44.6 %), 13. Functional rehabilitation (53.5 %) and 14. Neuro-psycho-motor recovery (50.5 %).

**Table 4:** The distribution of the subjects' options according to the score given on each item in the Kinetotherapeutic Subscale of QBTAP.

Kinetoterapeutic - Subscale – Likert Scale Points											
Items	Aquatic program	5 points		4 points		3 points		2 points		1 point	
		fi	%	fi	%	fi	%	fi	%	fi	%
1 Optimizing the general state of health	AG	50	49,5	30	29,7	16	15,8	3	3,0	2	2,0
	ATL	43	42,6	29	28,7	18	17,8	8	7,9	3	3,0
	TS	51	50,5	28	27,7	14	13,9	7	6,9	1	1,0
2. Stimulates blood circulation	AG	58	57,4	26	25,7	13	12,9	3	3,0	1	1,0
	ATL	42	41,6	26	25,7	23	22,8	6	5,9	4	4,0
	TS	56	55,4	23	22,8	16	15,8	5	5,0	1	1,0
3. Increases breathing capacity	AG	55	54,5	25	24,8	16	15,8	4	4,0	1	1,0
	ATL	36	35,6	27	26,7	26	25,7	9	8,9	3	3,0
	TS	52	51,5	22	21,8	21	20,8	4	4,0	2	2,0
4. Increases immunity and hardens the body	AG	41	40,6	35	34,7	21	20,8	4	4,0	–	–
	ATL	30	29,7	31	30,7	30	29,7	9	8,9	1	1,0
	TS	34	33,7	37	36,6	22	21,8	8	7,9	–	–
5. Improving joint mobility	AG	56	55,4	24	23,8	16	15,8	3	3,0	2	2,0
	ATL	41	40,6	25	24,8	24	23,8	10	9,9	1	1,0
	TS	57	56,4	20	19,8	17	16,8	6	5,9	1	1,0
6. Reduction of osteoporosis	AG	33	32,7	33	32,7	20	19,8	11	10,9	4	4,0
	ATL	21	20,8	31	30,7	29	28,7	15	14,9	5	5,0
	TS	40	39,6	26	25,7	21	20,8	11	10,9	3	3,0
7. Muscle relaxation	AG	30	29,7	29	28,7	27	26,7	11	10,9	4	4,0
	ATL	34	33,7	27	26,7	31	30,7	8	7,9	1	1,0
	TS	34	33,7	27	26,7	31	30,7	8	7,9	1	1,0
8. Postural correction and rehabilitation	AG	41	40,6	34	33,7	18	17,8	7	6,9	1	1,0
	ATL	26	25,7	32	31,7	29	28,7	12	11,9	2	2,0
	TS	66	65,3	14	13,9	13	12,9	7	6,9	1	1,0
9 Improvement of motor control	AG	40	39,6	37	36,6	18	17,8	4	4,0	2	2,0
	ATL	39	38,6	33	32,7	21	20,8	7	6,9	1	1,0
	TS	46	45,5	31	30,7	11	10,9	12	11,9	1	1,0
10. Maintaining homeostatic balance	AG	33	32,7	29	28,7	31	30,7	7	6,9	1	1,0
	ATL	24	23,8	35	34,7	30	29,7	10	9,9	2	2,0
	TS	33	32,7	36	35,6	23	22,8	8	7,9	1	1,0
11. Combating the effects of aging	AG	35	34,7	29	28,7	29	28,7	8	7,9	–	–
	ATL	31	30,7	24	23,8	34	33,7	12	11,9	–	–
	TS	35	34,7	26	25,7	27	26,7	13	12,9	–	–
12. Psychomotor recovery	AG	41	40,6	34	33,7	12	11,9	12	11,9	2	2,0
	ATL	44	43,6	33	32,7	17	16,8	6	5,9	1	1,0
	TS	45	44,6	31	30,7	19	18,8	4	4,0	2	2,0
13. Functional rehabilitation	AG	43	42,6	31	30,7	20	19,8	5	5,0	2	2,0
	ATL	37	36,6	32	31,7	21	20,8	7	6,9	4	4,0
	TS	54	53,5	24	23,8	18	17,8	4	4,0	1	1,0
14. Neuro-psycho-motor recovery	AG	41	40,6	31	30,7	19	18,8	6	5,9	4	4,0
	ATL	44	43,6	34	33,7	17	16,8	5	5,0	1	1,0
	TS	51	50,5	24	23,8	12	11,9	12	11,9	2	2,0
15. Improving metabolism	AG	51	50,5	27	26,7	18	17,8	4	4,0	1	1,0
	ATL	37	36,6	27	26,7	27	26,7	9	8,9	1	1,0
	TS	46	45,5	28	27,7	18	17,8	9	8,9	–	–
16. Combating obesity	AG	68	67,3	14	13,9	16	15,8	2	2,0	1	1,0
	ATL	42	41,6	21	20,8	27	26,7	8	7,9	3	3,0
	TS	50	49,5	21	20,8	21	20,8	7	6,9	2	2,0

AG – Aquagym program, ATL - Aquatic ludotherapy program, TS - therapeutic swimming program, fi -frequency.

In the kinetotherapeutic Subscale of QBTAP (Table 5), the highest percentage score was recorded by the therapeutic swimming program of 65.37 (39.9 %) points, followed by the Aquagym program with 65.32 (33.8 %) points, and the lowest score of 62.30 (32.3 %) by Aquatic ludotherapy program. For the Aquagym program, the items that recorded the highest average score were: 16. Fighting obesity with 4.44 points and 2. Stimulating blood circulation with 4.37 points, and the lowest score was recorded by items: 6. Reduction of osteoporosis and 7. Muscle relaxation. In the Aquatic ludotherapy program, the highest average scores were obtained by items: 14. Neuro-psycho-motor recovery with 4.13 points and 4. Increasing immunity and hardening the body, respectively by 12. Psychomotor recovery with 4.11 points, and the lowest average score was for item 6. Reduction of osteoporosis by 3.47 points. The therapeutic swimming program registered the highest average score for items: 8. Postural correction and rehabilitation with 4.35 points and for item 2. Stimulates blood circulation with 4.26 points, and the lowest score for item 11. Combating the effects aging by 3.82 points.

**Table 5:** Descriptive statistics of the kinetotherapeutic subscale of QBTAP for the three aquatic programs of the study.

Kinetotherapeutic - Subscale									
Programs	AG			ATL			TS		
Item	Σ	X	SD	Σ	X	SD	Σ	X	SD
1 Optimizing the general state of health	426,00	4,21	,95	404,00	4,00	1,09	424,00	4,19	,99
2. Stimulates blood circulation	440,00	4,35	,88	399,00	3,95	1,11	431,00	4,26	,96
3. Increases breathing capacity	432,00	4,27	,93	387,00	3,83	1,10	421,00	4,16	1,02
4. Increases immunity and hardens the body	416,00	4,11	,87	416,00	4,11	,87	400,00	3,96	,93
5. Improving joint mobility	432,00	4,27	,97	398,00	3,94	1,06	429,00	4,24	1,01
6. Reduction of osteoporosis	383,00	3,79	1,13	351,00	3,47	1,12	392,00	3,88	1,14
7. Muscle relaxation	373,00	3,69	1,12	398,00	3,94	1,12	388,00	3,84	1,01
8. Postural correction and rehabilitation	410,00	4,05	,97	371,00	3,67	1,04	440,00	4,35	1,01
9 Improvement of motor control	412,00	4,07	,95	405,00	4,00	,98	412,00	4,07	1,06
10. Maintaining homeostatic balance	389,00	3,85	,99	372,00	3,68	1,00	395,00	3.91	,98
11. Combating the effects of aging	394,00	3,90	,97	377,00	3,73	1,02	386,00	3.82	1,05
12. Psychomotor recovery	403,00	3,99	1,09	416,00	4,11	,96	416,00	4.11	,98
13. Functional rehabilitation	411,00	4,06	1,00	394,00	3,90	1,10	429,00	4,24	,95
14. Neuro-psycho-motor recovery	402,00	3,98	1,09	418,00	4,13	,93	413,00	4,08	1,13
15. Improving metabolism	426,00	4,21	,94	393,00	3,89	1,03	414,00	4,09	,99
16. Combating obesity	449,00	4,44	,89	394,00	3,90	1126	413,00	4,08	1,07
Total	6598	65,32	**-**	6293	62,30	**-**	6603	65,37	**-**
Percentage	33,8 %		**-**	32,3 %		**-**	33,9 %		**-**

AG – Aquagym program, ATL - Aquatic ludotherapy program, TS - therapeutic swimming program, Σ - sum, X – mean, SD−standard deviation.

The results in Table 6 of the psychological subscale of QBTAP highlight the fact that the highest frequency of 5-point Likert scale responses for the aquagym program (AG) compared to the other 2 programs, was recorded for the following items: 3. Improvement of self-esteem and self-image self (58.4 %), 7. Increasing adaptability to different environments (47.5 %), 9. Reducing anxiety (44.5 %) and 10. Optimizing motivations (39.6 %). For the aquatic ludotherapy program (ATL) compared to the other two programs, the highest percentage frequency of 5 points was recorded for the items: 1. Mental relaxation (61.4 %), 2. Combating stress (62.4 %). 4. Improving memory and attention (48.8 %), 5. Improving positive affectivity (45.5 %), 6. Formation of proactive behaviors (56.4 %), 8. Educational role (57.4 %) and 9. Reduced anxiety (44.5 %). The therapeutic swimming program (TS), compared to the other two programs, did not have the highest percentage frequency of maximum responses to any item.

**Table 6:** The distribution of the subjects' options according to the score given on each item in the Psychological Subscale of QBTAP.

Psychological - Subscale – Likert Scale Points											
Items	Aquatic program	5 points		4 points		3 points		2 points		1 point	
		fi	%	fi	%	fi	%	fi	%	fi	%
1. Mental relaxation	AG	49	48,5	27	26,7	17	16,8	6	5,9	2	2,0
	ATL	62	61,4	20	19,8	15	14,9	3	3,0	1	1,0
	TS	46	45,5	28	27,7	15	14,9	11	10,9	1	1,0
2. Combating stress	AG	56	55,4	23	22,8	15	14,9	6	5,9	1	1,0
	ATL	63	62,4	17	16,8	19	18,8	2	2,0	–	–
	TS	52	51,5	19	18,8	19	18,8	10	9,9	1	1,0
3. Improving self-esteem	AG	59	58,4	18	17,8	18	17,8	5	5,0	1	1,0
	ATL	34	33,7	32	31,7	25	24,8	8	7,9	2	2,0
	TS	46	45,5	22	21,8	19	18,8	12	11,9	2	2,0
4. Improving memory and attention	AG	38	37,6	30	29,7	23	22,8	7	6,9	3	3,0
	ATL	49	48,5	29	28,7	15	14,9	6	5,9	2	2,0
	TS	36	35,6	27	26,7	20	19,8	15	14,9	3	3,0
5. Improving positive affectivity	AG	40	39,6	26	25,7	20	19,8	12	11,9	3	3,0
	ATL	46	45,5	22	21,8	21	20,8	10	9,9	2	2,0
	TS	36	35,6	25	24,8	22	21,8	11	10,9	7	6,9
6. Formation of proactive behaviors	AG	29	28,7	39	38,6	22	21,8	10	9,9	1	1,0
	ATL	57	56,4	25	24,8	13	12,9	4	4,0	2	2,0
	TS	27	26,7	33	32,7	27	26,7	12	11,9	2	2,0
7. Increasing adaptability to different environments	AG	48	47,5	26	25,7	19	18,8	6	5,9	2	2,0
	ATL	47	46,5	28	27,7	18	17,8	6	5,9	2	2,0
	TS	41	40,6	31	30,7	20	19,8	6	5,9	3	3,0
8. Educational role	AG	50	49,5	25	24,8	19	18,8	5	5,0	2	2,0
	ATL	58	57,4	19	18,8	18	17,8	4	4,0	2	2,0
	TS	47	46,5	23	22,8	20	19,8	7	6,9	4	4,0
9. Reducing anxiety	AG	55	54,5	24	23,8	15	14,9	6	5,9	1	1,0
	ATL	55	54,5	25	24,8	14	13,9	5	5,0	2	2,0
	TS	47	46,5	22	21,8	18	17,8	11	10,9	3	3,0
10 Optimizing motivations	AG	40	39,6	32	31,7	20	19,8	7	6,9	2	2,0
	ATL	36	35,6	35	34,7	22	21,8	7	6,9	1	1,0
	TS	34	33,7	34	33,7	23	22,8	8	7,9	2	2,0

AG – Aquagym program, ATL - Aquatic ludotherapy program, TS - therapeutic swimming program, fi -frequency.

The arithmetic mean results allowed us to identify the most relevant items regarding the impact on mental functions according to the specifics of the three aquatic programs (Table 7). The Aquatic ludotherapy program recorded the highest average score 41.62 (34.3 %) points, followed by the Aquagym program with 40.81 (33.6 %) points, and the lowest average score was recorded by the Therapeutic swimming program 39.09 (32.1 %). For the Aquagym program, the items that recorded the highest average score were: 3. Improving self-esteem with 4.27 points and 2. Combating stress with 4.25 points, and the lowest score was recorded by the item 6. Formation of proactive behaviors with 3.84 points. The Aquatic ludotherapy program with the highest average score was obtained by the items: 2. Combating stress with 4.39 points and 1. Mental relaxation with 4.37 points, and the lowest average score by item 3. Improving self-esteem with 3.87 points. The therapeutic swimming program recorded the highest average score for the items: 2. Combating stress with 4.39 points and 1. Mental relaxation with 4.37 points, and the lowest score for item 6. Formation of proactive behaviors with 3.70 points.

**Table 7:** Descriptive statistics of the Psychological Subscale of QBTAP for the three aquatic programs of the study.

Psychological - Subscale									
Activities	AG			ATL			TS		
Items	Σ	X	SD	Σ	X	SD	Σ	X	SD
1. Mental relaxation	418,00	4,13	1,02	442,00	4,37	,914	410,00	4,05	1,06
2. Combating stress	430,00	4,25	,98	444,00	4,39	,861	414,00	4,09	1,09
3. Improving self-esteem	432,00	4,27	,99	391,00	3,87	1035	391,00	3,87	1,03
4. Improving memory and attention	396,00	3,92	1,07	420,00	4,15	1017	381,00	3,77	1,17
5. Improving positive affectivity	391,00	3,87	1,15	403,00	3,99	1117	375,00	3,71	1,25
6. Formation of proactive behaviors	388,00	3,84	,98	434,00	4,29	,975	374,00	3,70	1,05
7. Increasing adaptability to different environments	415,00	4,10	1,03	415,00	4,10	1028	404,00	4,00	1,05
8. Educational role	419,00	4,14	1,02	430,00	4,25	1016	405,00	4,00	1,14
9. Reducing anxiety	429,00	4,24	,98	429,00	4,24	1004	402,00	3,98	1,16
10 Optimizing motivations	404,00	4,00	1,02	401,00	3,97	,974	393,00	3,89	1,02
Total	4122	40,81	**-**	4209	41,67	**-**	3949	39,09	**-**
Percentage	33,6 %		**-**	34,3 %		**-**	32,1 %		**-**

AG – Aquagym program, ATL - Aquatic ludotherapy program, TS - therapeutic swimming program, Σ - sum, X – mean, SD−standard deviation.

**Fig. 1:**
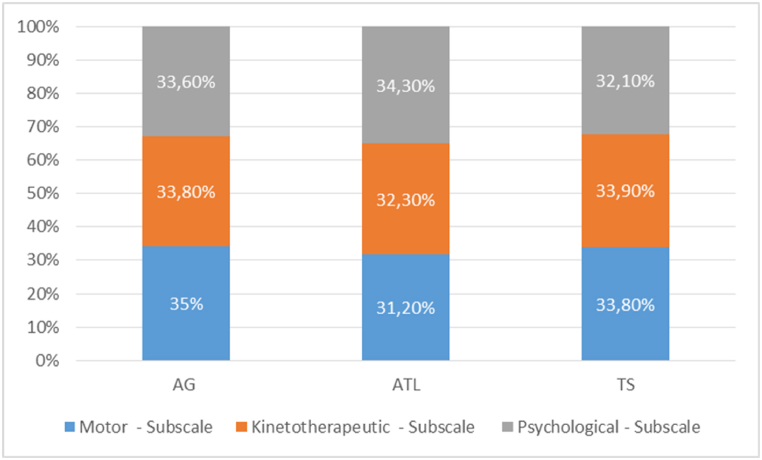
Percentages of average per subscales of QBTAP.

In the following graph, we have presented the percentage values of the arithmetic averages recorded for each subscale of the questionnaire, in order to highlight the differences between the aquatic programs (Fig. 1.).

## 4 Discussions

Our study focused on identifying the benefits of practicing three programs of aquatic activities on motor capacity, kinetotherapeutic effects and mental capacity from the perspective of physiotherapist students and master's degree students. Analyzing the results of the study, we find that the aquatic activities programs have multiple benefits, but they have a differentiated contribution depending on the capacities and the factors they target for improvement. Thus, from the point of view of improving the motor capacity, the recuperative aquagym (AG) program recorded the best result and the most specific items were identified that will be able to improve the motor capacity components compared to the other two programs. Physiokinetotherapists involved in the study considered that the therapeutic swimming program (TS) has the most benefits and the greatest impact on optimizing the recovery and rehabilitation process from a kinetotherapeutic perspective compared to the other two programs. Also, the results of the study reveal the fact that on the optimization of mental functions, aquatic ludotherapy programs have major benefits compared to the other two types of programs (AG and ATL).

The results of this study contribute to the expansion of the conceptual level of knowledge regarding the impact and benefits of the 3 aquatic activities programs on the development of motor capacity, the improvement of the kinetotherapeutic recovery and rehabilitation process and the optimization of human mental capacities. Previous studies substantiated the present study, and the results recorded by us complete the results and conclusions previously identified by experts in the field [[37], [38], [39]]. In previous studies, experts in the field approached the study of the effects of exercising in water from an interdisciplinary and multidimensional perspective, namely: harmonious physical development [40,41], neuromotor and functional recovery [42], in the fight against obesity [[43], [44], [45]], in reducing the effects of stress [[46], [47], [48]], in post-surgery rehabilitation processes and other medical problems [[49], [50], [51]], for recreational purposes [[52], [53], [54], [55]]. All these studies confirm the positive benefits of practicing physical activities in water, which also aligns with the results of our study.

The researchers claim that there is a growing concern in understanding the behavior of human biological systems in the aquatic environment, because the design of exercises and programs in this environment is increasing. Thus, it becomes important to understand the influences of the physical properties of the aquatic environment during exercise and the physiological adaptations that underlie them. Referring to the kinetotherapeutic and motor capacity, as a result of the studies carried out, the benefits identified were aimed at improving the health, the cardio-respiratory capacity, body composition [[56], [57], [58]], and motor capacity and physical fitness [[59], [60], [61]]. Regarding the physiological changes, it has been observed by several researchers that after an aquatic training program, the heart rate at rest has decreased, while maintaining an unchanged blood pressure value [62,63].

A series of studies have highlighted that the physical properties of water induce increases in motor coordinative qualities and mobility, the effects due to the buoyancy of the aquatic environment on the body cause decreases in the axial load of the spine, which allows the realization of certain movements, sometimes impossible to perform on the ground [[64], [65], [66]]. To adjust the body composition, some studies show significant decreases in the layer of adipose tissue in healthy sedentary people through aquatic training programs, obtaining the significant results after the application of 8-week programs [[67], [68], [69], [70]]. A series of studies have analyzed the influence of exercising in water regarding: motor capacity, health and cognitive functions, must focus on interdisciplinary approaches due to the complexity of the factors determined and the interrelationships between them [[71], [72], [73], [74]].

The present study analyzed the opinion of future specialist therapists regarding the benefits of aquatic programs whose results complement previous studies that carried out a quantitative evaluation of the impact of aquatic exercise on walking skills and balance in patients with multiple sclerosis or stroke [75,76], on pain therapy [77] (on the prevention of postpartum depression etc. [78]. We have not identified any study that analyzes the opinion of specialists or future specialists on the therapeutic effects of aquatic programs, but numerous studies have highlighted the effects of these programs on various medical conditions with an impact on physical and mental health [61,79].

The limits of the study: the social impact of these three types of aquatic programs was not analyzed; the negative effects of exercising in water from the perspective of subjects with chlorine allergies and fear of water were not analyzed; the proportion of female subjects who participated in the study was the majority; the limited duration of the implementation of aquatic programs. Strengths: analyzing three types of aquatic activities programs; analyzing the results from the point of view of the 3 subscales: motor, kinetotherapeutic and psychological; relatively large number of subjects involved in the study; the subjects are future specialists in physiotherapy; the subjects practiced these three types of aquatic programs during their academic studies.

The practical implications based on the most relevant results of the study: due to the multiple benefits of aquatic programs, we consider it appropriate to include different exercises of AG, ALT, TS in clinical recovery and rehabilitation protocols depending on the pathology of the patients and the specific aquatic facilities; presentation of various therapeutic swimming exercises to patients, so that they can be practiced independently in public swimming pools; recommending and implementing play therapy exercises in order to combat stress and negative psychological effects due to the major relaxing and recreational impact in patients with mental health problems; focusing and diversifying aquatic exercises according to the patients' pathology through the use of specific didactic materials; combining the three types of aquatic programs to increase their efficiency and attractiveness depending on the subject's preferences.

Future research directions: applying the questionnaire to different categories of patients with different pathologies who have benefited from the aquatic therapeutic programs; extending the programs in terms of duration in order to analyze and compare the results of this study with future studies; the quantitative and qualitative evaluation of the therapeutic and rehabilitation effects of aquatic programs on functional and motor capacity; the evaluation of the therapeutic impact through the application of combined programs of aquatic exercises on different categories of patients, interdisciplinary approaches to research on the therapeutic impact of aquatic programs on health and well-being etc.

## 5 Conclusions

The study highlights the beneficial effects of all three aquatic activity programs on improving motor capacity, optimizing the recovery and rehabilitation process from a kinetotherapeutic perspective and improving mental function. The comparative analysis of the results of the three types of programs reveals the fact that: aquagym (AG) programs have the greatest impact on improving motor capacity; aquatic ludotherapy programs (ATL) on improving function and mental capacity; the therapeutic swimming program (TS) has the most beneficial role on recovery and kinetotherapeutic rehabilitation.

The practical implications and future interdisciplinary approaches, based on the results of this study, will be able to be focused on: evaluation of the duration and complexity of the implementation in the process of practicing the three types of programs on the motor, kinetotherapeutic and mental components in different categories differentiated by age; on the typology of ailments and diseases; on the professional characteristics of the subjects; on the interaction with other professional, social, medical, educational, etc. factors.

## Funding

The authors declare that no financial support was received for the research.

## Data availability statement

The original contributions presented in the study are included in the article.

## Ethics statement

The study was conducted in accordance with the Declaration of Helsinki and approved no 24, on May 27, 2022, by the Review Board of the Physical Education Program of “G.E. Palade” University of Medicine, Pharmacy, Science, and Technology of Targu Mures, Romania.

## CRediT authorship contribution statement

**Dana Badau:** Writing – review & editing, Writing – original draft, Methodology, Investigation, Formal analysis, Data curation, Conceptualization. **Adela Badau:** Writing – review & editing, Writing – original draft, Investigation, Formal analysis, Data curation.

## Declaration of competing interest

The authors declare that they have no known competing financial interests or personal relationships that could have appeared to influence the work reported in this paper.

## References

[1] Aghaziarati A., Hu J., Guang-Song D. (2023). Mind-body interactions in chronic pain sufferers: a qualitative study on personality factors. Journal of Personality and Psychosomatic Research (JPPR).

[2] Navabinejad S., Mehdi R. (2023). Mind and body in sync: the fascinating field of psychophysiology in sports. Health Nexus.

[3] Jackson M., Kang M., Furness J., Kemp-Smith K. (2022). Aquatic exercise and mental health: a scoping review. Complement Ther Med.

[4] Lundin Å., Ekman I., Wallström S., Andréll P., Lundberg M. (2023). Suffering out of sight but not out of mind –interpreting experiences of sick leave due to chronic pain in a community setting: a qualitative study. BMJ Open.

[5] Waller B., Lambeck J., Daly D. (2009). Therapeutic aquatic exercise in the treatment of low back pain: a systematic review. Clin. Rehabil..

[6] Seywert A.J., Tappy L., Gremion G., Giusti V. (2002). Effect of a program of moderate physical activity on mental stress-induced increase in energy expenditure in obese women. Diabetes Metab..

[7] Pennick V.E., Young G. (2007). Interventions for preventing and treating pelvic and back pain in pregnancy. Cochrane Database Syst. Rev..

[8] Gutenbrunner C., Briest J., Egen C. (2021). "Fit for work and life": an innovative concept to improve health and work ability of employees, integrating prevention, therapy and rehabilitation. J. Rehabil. Med..

[9] Ubago-Guisado E., Sánchez Sánchez J., Vila Maldonado S., Gallardo L. (2019). Effects of Zumba® and aquagym on bone mass in inactive middle-aged women. Medicina (Kaunas).

[10] Baena-Beato P.Á., Artero E.G., Arroyo-Morales M., Robles-Fuentes A., Gatto-Cardia M.C., Delgado-Fernández M. (2014). Aquatic therapy improves pain, disability, quality of life, body composition and fitness in sedentary adults with chronic low back pain. A controlled clinical trial. Clin. Rehabil..

[11] Becker B.E. (2020). Aquatic therapy in contemporary neurorehabilitation: an update. PM&R.

[12] Gappmaier E., Lake W., Nelson A.G., Fisher A.G. (2006). Aerobic exercise in water versus walking on land: effects on indices of fat reduction and weight loss of obese women. J. Sports Med. Phys. Fit..

[13] Campos-Mesa M.C., Del Castillo O., Montiel-Ortega P. (2015). Efectos de un programa de fitness acuático sobre la condición física en mujeres postmenopáusicas. Journal of Sport and Health Research.

[14] Badau A., Ungur R.N., Badau D. (2015). Influence of water gymnastics on strength development. Palestrica of the Third Millennium Civilization & Sport.

[15] Torlaković A., Muftić M., Avdić D., Kebata R. (2013). Effects of the combined swimming, corrective and aqua gymnastics programme on body posture of preschool age children. Journal of Health Sciences.

[16] Badau D., Badau A. (2015). The Influence of various types of water gymnastics upon the exercise capacity. International Journal of Sport Culture and Science.

[17] Bădescu V. Activitati si jocuri acvatice distractive. http://activitatifiziceadaptate.ro/wp-content/uploads/2015/01/ACTIVIT%C4%82%C8%9AI-%C8%98I-JOCURI-ACVATICE-DISTRACTIVE-Conf.-univ.-dr.-Badescu-Victor.pdf.

[18] González-Ravé J.M., Turner A.P., Phillips S.M. (2020). Adaptations to swimming training in athletes with down's syndrome. Int. J. Environ. Res. Publ. Health.

[19] Reis D.T.F., Pereira R.R., da Silva R.A. (2023). Influence of physiotherapy in the treatment of children with autism spectrum disorder. Seven Editora.

[20] Puspitosari A. (2022). Play therapy modified ludo games decreasing hyperactivity, impulsivity, and inattention in children with attention deficit and hiperactivity disorder. Jurnal Keterapian Fisik.

[21] Barker A.L., Talevski J., Morello R.T., Brand C.A., Rahmann A.E., Urquhart D.M. (2014). Effectiveness of aquatic exercise for musculoskeletal conditions: a meta-analysis. Arch. Phys. Med. Rehabil..

[22] Schaefer S.Y., Louder T.J., Foster S., Bressel E. (2016). Effect of water immersion on dual‐task performance: implications for aquatic therapy. Physiother. Res. Int..

[23] Becker B.E. (2009). Aquatic therapy: scientific foundations and clinical rehabilitation applications. Pm&r.

[24] Lim J.Y., Tchai E., Jang S.N. (2010). Effectiveness of aquatic exercise for obese patients with knee osteoarthritis: a randomized controlled trial. Pm&r.

[25] García I., Molina-Molina M., Arrillaga B., Javierre C., Viscor G. (2022). Swimming exercise for patients with long-term respiratory post COVID-19 complications: further thinking on the pulmonary rehabilitation. Arch. Bronconeumol..

[26] Gojkovic Z., Ivancevic T., Jovanovic B. (2019). Biomechanical model of swimming rehabilitation after hip and knee surgery. J. Biomech..

[27] Su Y., Chen Z., Xie W. (2020). Swimming as treatment for osteoporosis: a systematic review and meta-analysis. BioMed Res. Int..

[28] Douris P., Southard V., Varga C., Schauss W., Gennaro C., Reiss A. (2003). The effect of land and aquatic exercise on balance scores in older adults. J. Geriatr. Phys. Ther..

[29] Wang T.J., Lee S.C., Liang S.Y., Tung H.H., Wu S.F.V., Lin Y.P. (2011). Comparing the efficacy of aquatic exercises and land‐based exercises for patients with knee osteoarthritis. J. Clin. Nurs..

[30] Jackson J., Karavatas S.G., Greene R.A., Brown-White P., Burnett C.A. (2014). Systematic review of the benefits of land-based exercise compared to aquatic exercise in increasing cardiovascular endurance. Journal of the National Society of Allied Health.

[31] Batterham S.I., Heywood S., Keating J.L. (2011). Systematic review and meta-analysis comparing land and aquatic exercise for people with hip or knee arthritis on function, mobility and other health outcomes. BMC Muscoskel. Disord..

[32] Sanders M.E., Islam M.M., Naruse A., Takeshima N., Rogers M.E. (2016). Aquatic exercise for better living on land: impact of shallow-water exercise on older Japanese women for performance of activities of daily living (ADL). Int. J. Aquat. Res. Educ..

[33] Abadi F.H., Choo L.A., Sankaravel M., Mondam S. (2018). A comparative study of water and land based exercises training program on stability and range of motion. Int J Adv Res Technol.

[34] Bergamin M., Ermolao A., Tolomio S., Berton L., Sergi G., Zaccaria M. (2013). Water-versus land-based exercise in elderly subjects: effects on physical performance and body composition. Clin. Interv. Aging.

[35] Barbosa T.M., Garrido M.F., Bragada J. (2007). Physiological adaptations to head-out aquatic exercises with different levels of body immersion. J. Strength Condit Res..

[36] Matsui T., Onodera S. (2013). Cardiovascular responses in rest, exercise, and recovery phases in water immersion. The Journal of Physical Fitness and Sports Medicine.

[37] Raffaelli C., Milanese C., Lanza M., Zamparo P. (2016). Water-based training enhances both physical capacities and body composition in healthy young adult women. Sport Sci. Health.

[38] Takeshima N., Rogers M.E., Watanabe E., Brechue W.F., Okada A., Yamada T., Hayano J. (2002). Water-based exercise improves health-related aspects of fitness in older women. Med. Sci. Sports Exerc..

[39] Meredith-Jones K., Waters D., Legge M., Jones L. (2011). Upright water-based exercise to improve cardiovascular and metabolic health: a qualitative review. Compl. Ther. Med..

[40] Holovkina V.V., Salnykova S.V., Sulyma A.S., Brezdeniuk O.Y., Korolchuk A.P., Nesterova S.Y. (2018). Effect of swimming with the use of aqua fitness elements and interval hypoxic training on the physical fitness of boys aged 11-12 years. Pedagogics, psychology, medical-biological problems of physical training and sports.

[41] Stan E.A. (2012). The benefits of aerobic aquatic gymnastics on overweight children. Palestrica of the Third Millennium Civilization & Sport.

[42] Elnaggar R.K., Alghadier M., Abdrabo M.S., Abonour A.A. (2022). Effect of a structured aqua-plyometric exercise program on postural control and functional ability in children with hemiparetic cerebral palsy: a two-arm randomized controlled trial. NeuroRehabilitation.

[43] Lee B.A., Oh D.J. (2014). The effects of aquatic exercise on body composition, physical fitness, and vascular compliance of obese elementary students. Journal of exercise rehabilitation.

[44] Greene N.P., Lambert B.S., Greene E.S., Carbuhn A.F., Green J.S., Crouse S.F. (2009). Comparative efficacy of water and land treadmill training for overweight or obese adults. Med. Sci. Sports Exerc..

[45] Kantyka J., Herman D., Roczniok R., Kuba L. (2015). Effects of aqua aerobics on body composition, body mass, lipid profile, and blood count in middle-aged sedentary women. Hum. Mov..

[46] Kravitz L., Mayo J.J. (2021). The physiological effects of aquatic exercise. Int. J. Med..

[47] Marzouki H., Soussi B., Selmi O., Hajji Y., Marsigliante S., Bouhlel E., Knechtle B. (2022). Effects of aquatic training in children with autism spectrum disorder. Biology.

[48] Ali K.M., El Gammal E.R., Eladl H.M. (2021). Effect of aqua therapy exercises on postmastectomy lymphedema: a prospective randomized controlled trial. Annals of Rehabilitation Medicine.

[49] Lee C.H., Kim I.H. (2021). Aquatic exercise and land exercise treatments after total knee replacement arthroplasty in elderly women: a comparative study. Medicina.

[50] Buckthorpe M., Pirotti E., Della Villa F. (2019). Benefits and use of aquatic therapy during rehabilitation after ACL reconstruction-a clinical commentary. International journal of sports physical therapy.

[51] Iranpour A., Gorbanian B., Bolboli L., Valizadehorang A., Azarian S. (2020). The influence of aqua aerobic exercise on cardiac autonomic function and blood pressure in college male students. Journal of Advanced Sport Technology.

[52] Petrenko N.V., Loza T.A. (2014). Model of recreational and training sessions based on the use of funds aqua professionally applied in the preparation of students of economics. Physical education of students.

[53] Drohomirecka A., Wojciuszkiewicz J. (2016). Opinion about exercises in water and lifestyle of women attending aqua aerobics classes. Central European Journal of Sport Sciences and Medicine.

[54] Al Haddad H., Parouty J., Buchheit M. (2012). Effect of daily cold water immersion on heart rate variability and subjective ratings of well-being in highly trained swimmers. Int. J. Sports Physiol. Perform..

[55] Adsett J.A., Mudge A.M., Morris N., Kuys S., Paratz J.D. (2015). Aquatic exercise training and stable heart failure: a systematic review and meta-analysis. Int. J. Cardiol..

[56] Guimarães A.L.A., Gomes-Neto M., Conceição L.S.R., Saquetto M.B., Gois C.O., Carvalho V.O. (2023). Water-based exercises on peak oxygen consumption, exercise time, and muscle strength in patients with coronary artery disease: a systematic review with meta-analysis. Cardiovasc Ther.

[57] Mocanu G.D. (2023). Analysis of differences in Muscle Power for female university students majoring in sports according to BMI levels. Balneo & PRM Research Journal.

[58] Colado J.C., Tella V., Triplett N.T., González L.M. (2009). Effects of a short-term aquatic resistance program on strength and body composition in fit young men. J Strength Cond Res.

[59] Mocanu G.D., Onu I. (2022). The influence of specialization and the level of physical activism on leisure options for students of the Faculty of Physical Education and Sports. Balneo and PRM Research Journal.

[60] Li C., Khoo S., Adnan A. (2017). Effects of aquatic exercise on physical function and fitness among people with spinal cord injury: a systematic review. Medicine.

[61] Silva L.A.D., Menguer L.D.S., Doyenart R. (2022). Effect of aquatic exercise on mental health, functional autonomy, and oxidative damages in diabetes elderly individuals. Int. J. Environ. Health Res..

[62] Jackson M., Kang M., Furness J., Kemp-Smith K. (2022). Aquatic exercise and mental health: a scoping review. Complement Ther Med.

[63] Hakim R.M., Ross M.D., Runco W., Kane M.T. (2017). A community-based aquatic exercise program to improve endurance and mobility in adults with mild to moderate intellectual disability. J Exerc Rehabil.

[64] Wang T.J., Belza B., Elaine Thompson F., Whitney J.D., Bennett K. (2007). Effects of aquatic exercise on flexibility, strength and aerobic fitness in adults with osteoarthritis of the hip or knee. J. Adv. Nurs..

[65] Rahmann A.E., Brauer S.G., Nitz J.C. (2009). A specific inpatient aquatic physiotherapy program improves strength after total hip or knee replacement surgery: a randomized controlled trial. Arch. Phys. Med. Rehabil..

[66] Greene N.P., Martin S.E., Crouse S.F. (2012). Acute exercise and training alter blood lipid and lipoprotein profiles differently in overweight and obese men and women. Obesity.

[67] Phillips V.K., Legge M., Jones L.M. (2008). Maximal physiological responses between aquatic and land exercise in overweight women. Med. Sci. Sports Exerc..

[68] Ogonowska-Slodownik A., Richley Geigle P., Morgulec-Adamowicz N. (2020). Head-out water-based protocols to assess cardiorespiratory fitness-systematic review. Int. J. Environ. Res. Publ. Health.

[69] Kwok M.M.Y., Poon E.T.C., Ng S.S.M., Lai M.C.Y., So B.C.L. (2022). Effects of aquatic versus land high-intensity interval training on acute cardiometabolic and perceptive responses in healthy young women. Int. J. Environ. Res. Publ. Health.

[70] Oral O., Tatlibal P., Stavropoulou E. (2021). Effects of aquatic exercise in the treatment of obesity. Biomed. J. Sci. Tech. Res.

[71] Hogea T., Suciu B.A., Ivanescu A.D., Carasca C., Chinezu L., Arbanasi E.M., Russu E., Kaller R., arbanasi E.M., Muresan A.V., Radu C.C. (2023). Increased epicardial adipose tissue (EAT), left coronary artery plaque morphology, and valvular atherosclerosis as risks factors for sudden cardiac death from a forensic perspective. Diagnostics.

[72] Martoma A. (October 4-6, 2010). 9th WSEAS International Conference on Education and Educational Technology (Edu’10).

[73] Stoica M., Stoica A., Gozu B. (2012). Study on the importance of the athletic exercises in preventing and combating overweight and obesity in children. Studia Universitatis Babes-Bolyai Educatio Artis Gymnasticae.

[74] Nissim M., Ram-Tsur R., Zion M., Mevarech Z., Ben-Soussan T.D. (2014). Effects of aquatic motor activities on early childhood cognitive and motor development. Open J. Soc. Sci..

[75] Hao Z., Zhang X., Chen P. (2022). Effects of different exercise therapies on balance function and functional walking ability in multiple sclerosis disease patients—a network meta-analysis of randomized controlled trials. Int. J. Environ. Res. Publ. Health.

[76] Gu X., Zeng M., Cui Y., Fu J., Li Y., Yao Y., Shen F., Sun Y., Wang Z., Deng D. (2023). Aquatic strength training improves postural stability and walking function in stroke patients. Physiother. Theory Pract..

[77] Pérez de la Cruz S. (2017). Effectiveness of aquatic therapy for the control of pain and increased functionality in people with Parkinson's disease: a randomized clinical trial. Eur. J. Phys. Rehabil. Med..

[78] Aguilar-Cordero M.J., Sánchez-García J.C., Rodriguez-Blanque R., Sánchez-López A.M., Mur-Villar N. (2019). Moderate physical activity in an aquatic environment during pregnancy (SWEP study) and its influence in preventing postpartum depression. J. Am. Psychiatr. Nurses Assoc..

[79] Silva L.A.D., Tortelli L., Motta J., Menguer L., Mariano S., Tasca G., Silveira G.B., Pinho R.A., Silveira P.C.L. (2019). Effects of aquatic exercise on mental health, functional autonomy and oxidative stress in depressed elderly individuals: a randomized clinical trial. Clinics.

